# Nanoparticle Properties Modulate Their Attachment and Effect on Carrier Red Blood Cells

**DOI:** 10.1038/s41598-018-19897-8

**Published:** 2018-01-25

**Authors:** Daniel C. Pan, Jacob W. Myerson, Jacob S. Brenner, Priyal N. Patel, Aaron C. Anselmo, Samir Mitragotri, Vladimir Muzykantov

**Affiliations:** 1 Department of Pharmacology and Center for Translational Targeted Therapeutics and Nanomedicine, Perelman School of Medicine, University of Pennsylvania, Philadelphia, Pennsylvania 19104 United States; 2 Pulmonary and Critical Care Division, University of Pennsylvania, Philadelphia, Pennsylvania 19104 United States; 3 Division of Pharmacoengineering and Molecular Pharmaceutics, Eshelman School of Pharmacy, University of North Carolina at Chapel Hill, Chapel Hill, North Carolina 27599 United States; 4 School of Engineering and Applied Sciences, Harvard University, Cambridge, Massachusetts, 02138 United States

## Abstract

Attachment of nanoparticles (NPs) to the surface of carrier red blood cells (RBCs) profoundly alters their interactions with the host organism, decelerating NP clearance from the bloodstream while enabling NP transfer from the RBC surface to the vascular cells. These changes in pharmacokinetics of NPs imposed by carrier RBCs are favorable for many drug delivery purposes. On the other hand, understanding effects of NPs on the carrier RBCs is vital for successful translation of this novel drug delivery paradigm. Here, using two types of distinct nanoparticles (polystyrene (PSNP) and lysozyme-dextran nanogels (LDNG)) we assessed potential adverse and sensitizing effects of surface adsorption of NPs on mouse and human RBCs. At similar NP loadings (approx. 50 particles per RBC), adsorption of PSNPs, but not LDNGs, induces RBCs agglutination and sensitizes RBCs to damage by osmotic, mechanical and oxidative stress. PSNPs, but not LDNGs, increase RBC stiffening and surface exposure of phosphatidylserine, both known to accelerate RBC clearance *in vivo*. Therefore, NP properties and loading amounts have a profound impact on RBCs. Furthermore, LDNGs appear conducive to nanoparticle drug delivery using carrier RBCs.

## Introduction

Optimizing pharmacokinetics (PK) is an important element of design of vascular drug delivery systems^1–3^. A number of methods have been developed to prolong circulation time of nanocarriers/nanoparticles (NPs) and avoid uptake by the reticuloendothelial system (RES), such as PEG surface coatings, modification of NP shape (e.g. elongated), and increased mechanical flexibility of NPs^4–25^. More recently, improved circulation time was achieved through the adsorption of NPs onto the surface of red blood cells (RBCs)^21,22,26–32^.

The attempts to coopt RBCs, natural carriers for many endogenous compounds, to carry drugs span several decades^33–36^. RBCs, the most abundant cellular constituent of the blood (>99%), have many features of ideal drug carriers, including their bioavailability, biocompatibility, and longevity in circulation (approximately 45 days in mice and 120 days in humans). Beyond prolonging an adsorbed NP’s lifetime in the bloodstream, RBCs offer mechanisms for masking attached cargo compounds by the glycocalyx^37–40^ and for cargo delivery to vascular targets including thrombi, immune cells, macrophages, endothelial cells, and the RES sinuses^41–44^.

There have been many studies investigating blood-biomaterial interactions. Foreign materials that interact with bodily components such as platelets and RBCs can provoke complement activation, inflammation, and thrombosis^45^. Platelet activation and aggregation can be induced by different materials, such as TiO_2_, in the presence of Ca^2+^, collagen, fibronectin, and poly-L-lysine^46,47^. For RBCs, detrimental interactions such as aggregation, crenation, and hemolysis suggest incompatibility. Previous studies showed that non-covalent adsorption of rigid polymeric NPs (200 nm polystyrene spherical beads used as model drug delivery vehicles) to naive mouse RBCs altered the behavior of the resultant RBC-NP complexes upon intravenous administration in naive mice. The circulation time of RBC-bound NPs was remarkably longer than that of free NPs. Importantly, adsorption of NPs onto RBCs at ratios up to 50:1 did not accelerate elimination of the carrier RBC^29^. Furthermore, RBC adsorption enabled the transfer of reversibly associated NPs to the pulmonary vasculature^29^. More recently, and more comforting in translational terms, it was reported that human RBCs are generally less fragile and more resistant to adverse effects of NPs than mouse RBCs^48^. This previous body of work leveraged the use of a model NP type, and the results strongly suggests that attachment of NPs to the surface of RBCs can lead to many advantages in NP drug delivery.

However, little is known about changes caused to RBCs by NPs, as a result of either intentional adsorption on RBCs or unintentional interactions. The RBC plasma membrane is characterized by a unique combination of high mechanical flexibility necessary to squeeze through the microvasculature, and physical sturdiness needed to survive high hydrodynamic stresses in circulation, especially in the heart and aorta^49–52^. Senescent, infected (e.g., by malaria plasmodium), genetically impaired (e.g., in sickle cell disease), or artificially modified RBCs are not as resistant to damaging factors, leading to membrane disruption and release of the RBC’s main protein cargo, hemoglobin^48,52–62^. Previous studies have indicated that adsorption of NPs onto murine RBCs is not overtly detrimental for short periods of time (<8 h) and that RBCs tolerate carriage of rigid NPs at loading doses providing attachment of up to an average of about 50 NP per RBC *in vitro* without inducing hemolysis^48^. However, the adsorption of polystyrene beads onto RBCs at ratios of 50:1 or greater causes their agglutination *in vitro*^48^. In addition, polystyrene beads have been found to alter membrane structures in molecular stimulations^63,64^.

In the present study, we aimed to elucidate the role that biomaterial properties have in regulating interactions with RBCs. RBC damage induced *in vitro* by different NPs was assessed using assays developed in our previous work^48^. We have demonstrated that the adsorption of polystyrene beads (PSNP) induces negative effects on RBCs, whereas the adsorption of NPs made from soft biodegradable materials, lysozyme-dextran nanogels (LDNGs), does not induce negative effects on RBCs, strongly suggesting that NP properties directly relate to biocompatibility. LDNGs, composed of biodegradable components with validated *in vivo* use^65,66^, have not been extensively investigated as a NP for cell-mediated delivery. These results are important and encouraging in the context of both understanding how distinct materials interact with RBCs and in the potential utility of LDNGs as a cargo to be delivered by RBCs.

## Results

### Characterization of NPs

Table 1 lists the basic characteristics of the NPs employed in the study. As determined by dynamic light scattering, the diameter of PSNP and LDNG was 171 ± 3.0 nm and 268 ± 9.6 nm, respectively, whereas the size of their IgG-conjugated counterparts was 239 ± 1.2 nm and 279 ± 3.8 nm, respectively. The NPs showed suitable size distribution, with PDI values of 0.023 and 0.12 for PSNP and LDNG, respectively, whereas the PDI values of their IgG-conjugated counterparts was 0.074 and 0.177, respectively. The particles exhibited distinct surface charges; the zeta potential was strongly negative for PSNP and close to neutral for LDNG (33.27 ± 1.05 vs −0.42 ± 0.09 mV, respectively). The conjugation of IgG onto PSNP and LDNGs increased the zeta potential of both particles (−3.98 ± 0.76 mV vs 0.43 ± 0.16 mV). The larger PSNP had a diameter of 319 ± 1.6 nm (PDI of 0.069) and had a zeta potential that was strongly negative (−54.83 ± 0.23 mV) (Supplemental Table 1).

**Table 1:** Size and charge of unmodified and IgG-Nanoparticles.

	PSNP	IgG-PSNP	LDNGs	IgG-LDNGs
Size (nm)	171.1 ± 3.0	239.0 ± 1.2	268.2 ± 9.6	278.7 ± 3.8
PDI	0.023 ± 0.01	0.074 ± 0.02	0.117 ± 0.02	0.177 ± 0.04
Zeta Potential (mV)	−33.27 ± 1.05	−3.98 ± 0.76	−0.42 ± 0.09	0.43 ± 0.16

### Adsorption of NP to RBC

**Figure 1:**
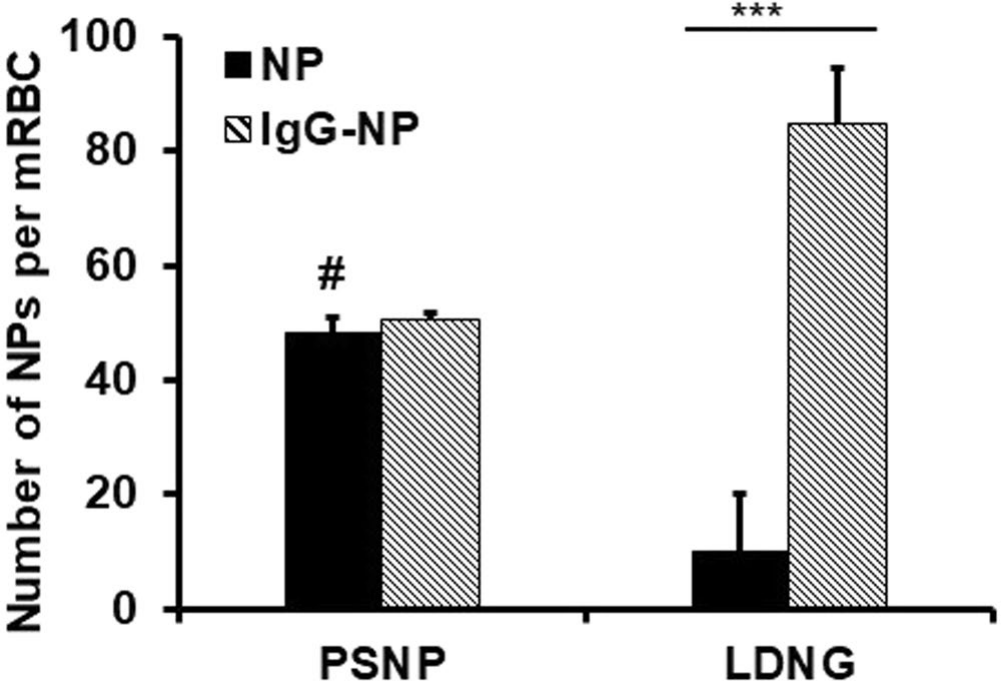
The number of nanoparticles adsorbed onto murine red blood cells. Number of unmodified or IgG coated polystyrene beads (PSNP) as well as unmodified or IgG coated lysozyme dextran nanogels (LDNGs) adsorbed per murine RBC at NP:RBC loading ratio of 200:1. Values are means (n = 4–7) ± SEM. (***P < 0.001 vs. unmodified NP). ^#^Represents extrapolation via least squares linear regression of number of particles adsorbed onto murine RBC, as described in previous work^29^.

Table 2 and Fig. 1 quantify binding of radiolabeled NP formulations to washed RBCs. Pristine PSNP bound to murine RBCs at 4 times the rate of LDNG particles. This enhanced binding can be explained by the differences in surface charge, as shown in Table 1.

**Table 2:** Attachment of Nanoparticles to RBC.

	Murine RBCs		Human RBCs	
	% Adsorption Efficiency	^#^NP/RBC	% Adsorption Efficiency	^#^NP/RBC
PSNP	24.0 ± 1.5	48.0 ± 3.0*	NA	NA
IgG-PSNP	25.3 ± 5.0	50.5 ± 10.0	26.7 ± 5.7	53.5 ± 11.5
LDNGs	5.1 ± 0.3	10.1 ± 1.0	6.1 ± 2.8	12.2 ± 5.5
IgG-LDNGs	48.5 ± 5.0	84.7 ± 10.0	22.2 ± 2.7	44.3 ± 5.4
IgG alone	0.7 ± 0.1	NA	1.7 ± 0.4	NA

Adsorption of various nanoparticles were at NP:RBC ratio of 200:1. *Represents extrapolation via least squares linear regression of number of particles adsorbed onto murine RBC, as described in previous work^29^. NA represents values not assessed.

Next, we tested whether loading to RBC can be modulated by coating the surface of NPs with IgG, a generic model protein widely used as a control in drug delivery applications and also readily encountered in blood. Of note, radiolabeled IgG itself didn’t bind substantially to either murine or human RBCs (Table 2); however, conjugating NPs with IgG altered NP binding to RBCs. In particular, IgG-conjugated LDNG (IgG-LDNG) bound to RBCs at nearly ten times the rate of the non-IgG LDNG counterpart. The effect of IgG conjugation on PSNP prior to binding to RBC was not as pronounced and exhibited no statistical differences. As a result, murine RBC carried approximately 90 NPs per RBC in the case of IgG-LDNG versus approximately 50 NPs per RBC in the case of IgG-PSNP. In the case of human RBC, both IgG-LDNG and IgG-PSNP showed similar RBC binding, approaching 50 particles per cell.

### Agglutination of RBC by PSNP and LDNG particles

**Figure 2:**
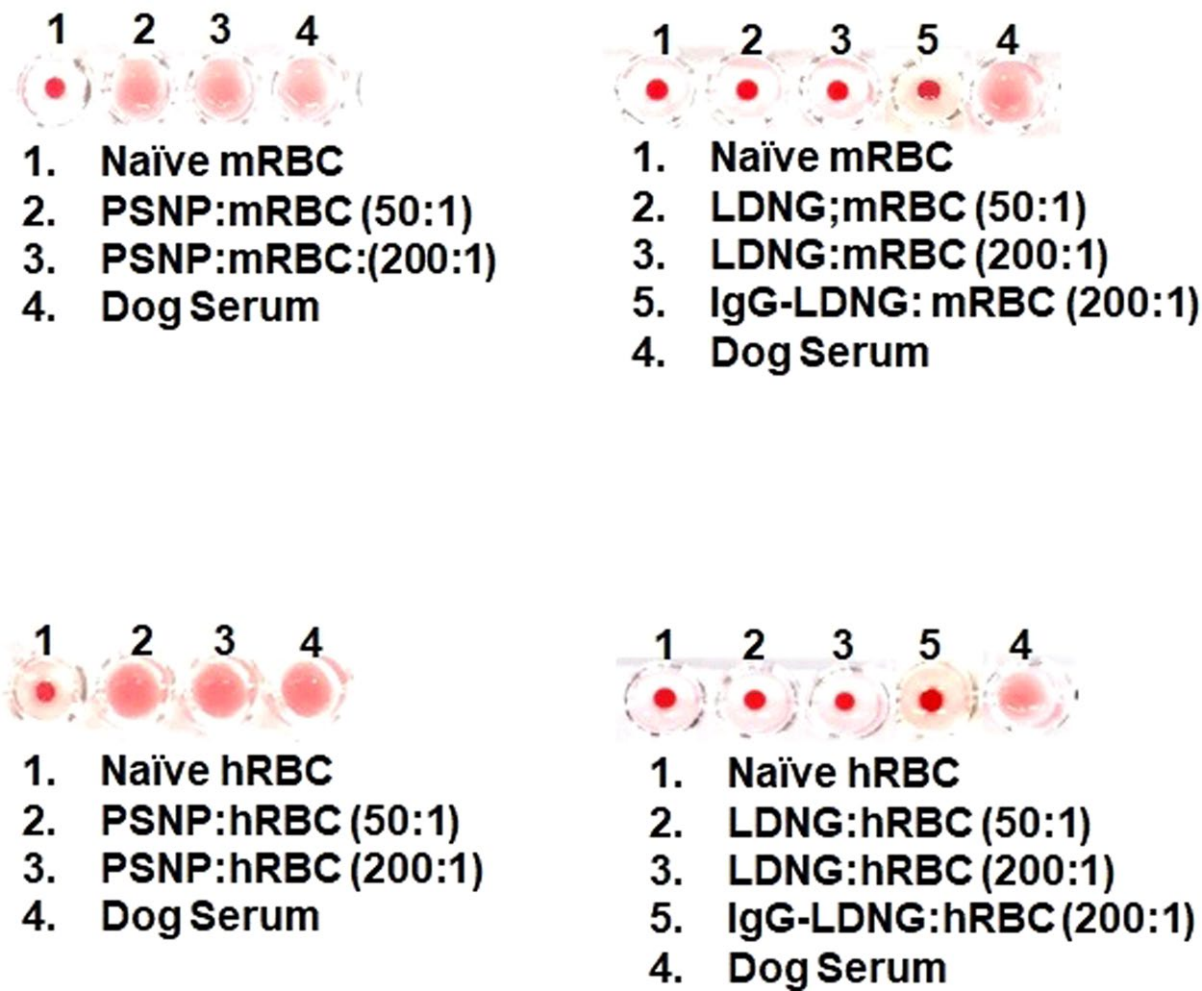
Agglutination of red blood cells with nanoparticles adsorbed onto their surface. Agglutination was visualized using U-shaped wells containing murine and human red blood cells. Treatment of RBCs with dog serum was used as a positive control for agglutination.

PSNP of two diameters (~200 nm and ~300 nm), incubated with either murine or human RBC at NP:RBC loading ratio of 50:1, cause RBC aggregation. This is demonstrated through the use of a standard agglutination assay in U-shaped microwells (Fig. 2, Supplementary Fig. S2) and in an independent microscopic examination (Supplemental Fig. S1). In contrast, neither LDNG nor IgG-LDNG caused significant RBC agglutination even at 4-fold higher NP:RBC ratios (Fig. 2).

### Sensitivity of RBC:NP to Osmotic Stress

**Figure 3:**
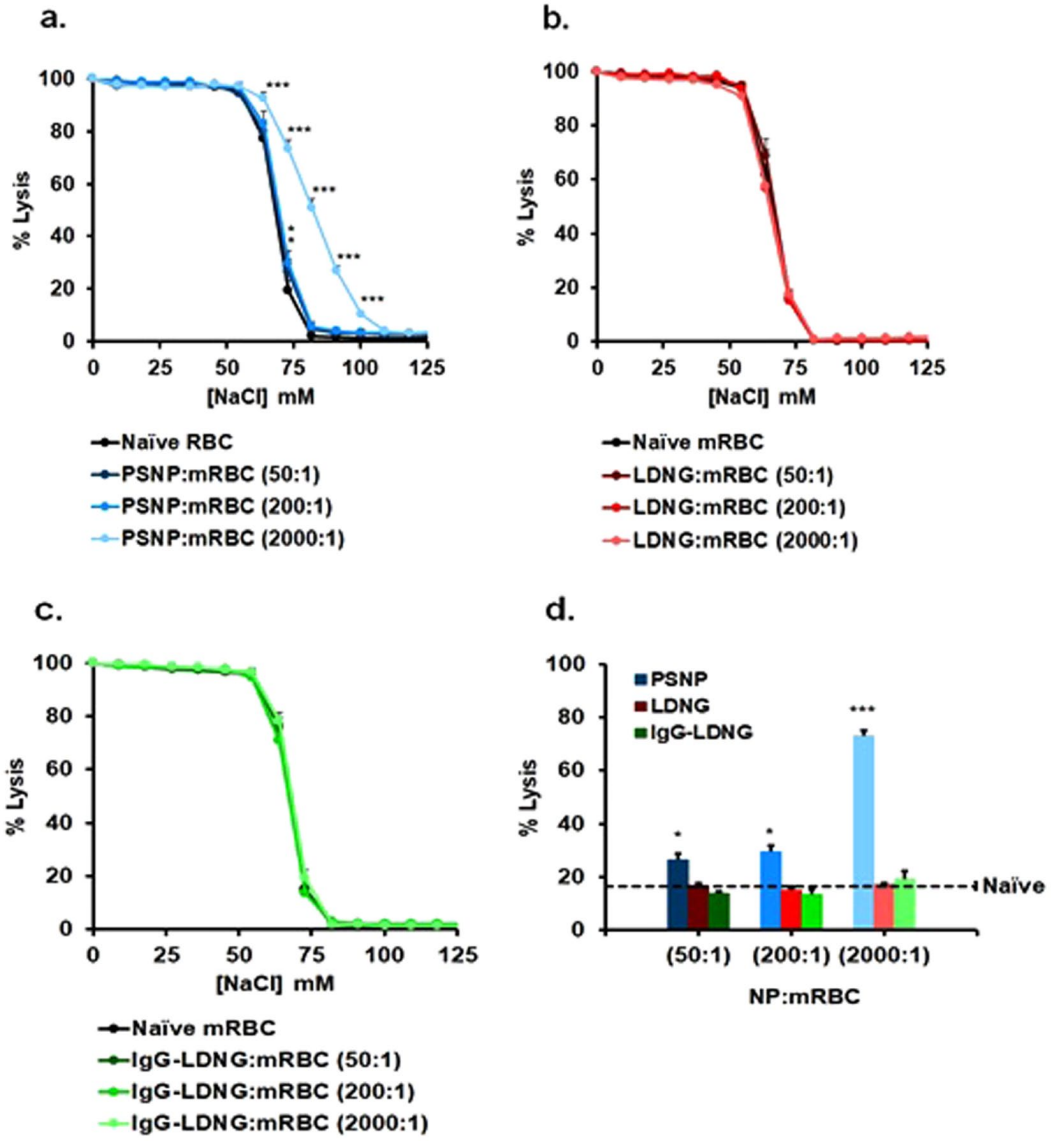
Osmotic fragility of murine red blood cells with adsorbed nanoparticles. Representative osmotic fragility curves for NP:murine RBC at NP:RBC ratio 50:1, 200:1, or 2000:1 immediately after exposure to different [NaCl] following adsorption of PSNP (**a**), LDNGs (**b**), or IgG-LDNGs (**c**). Percent hemolysis for different NP:RBC ratios at 73 mM NaCl (**d**). Values are means (n = 4–6) ± SEM. (*P < 0.05; ***P < 0.001 vs naïve RBC).

Next, we tested the effect of NP binding on RBC sensitivity to osmotic lysis. Binding of PSNP to murine RBCs aggravates hemolysis in hypotonic NaCl concentrations ranging from ~100 to ~50 mM (Fig. 3a) at PSNP:RBC loading ratio of 2000:1, whereas neither LDNG nor IgG-LDNG particles caused this effect (Fig. 3b and c). At the rate-limiting osmolality (~75 mM NaCl), the sensitization of RBCs to osmotic lysis was detectable at 200 nm PSNP:RBC loading ratios 50:1, 200:1 (~30% lysis vs. ~20% for naïve RBCs), and 2000:1 (~75% lysis), while LDNGs did not detectably enhance lysis even at the excessively high ratio of 2000:1 (Fig. 3d). Additionally, larger PSNP particles (300 nm diameter) at PSNP:RBC loading ratio of 2000:1 aggravated osmotic lysis in a similar fashion to 200 nm PSNPs (Supplemental Fig. S2). In accordance with previous observations, human RBCs are more resistant to osmotic lysis and therefore neither PSNPs nor LDNGs caused significant lysis even at NP:RBC loading ratios of 2000:1 (Supplemental Fig. S3).

### Sensitivity of NP-carrying RBC to shear stress

**Figure 4:**
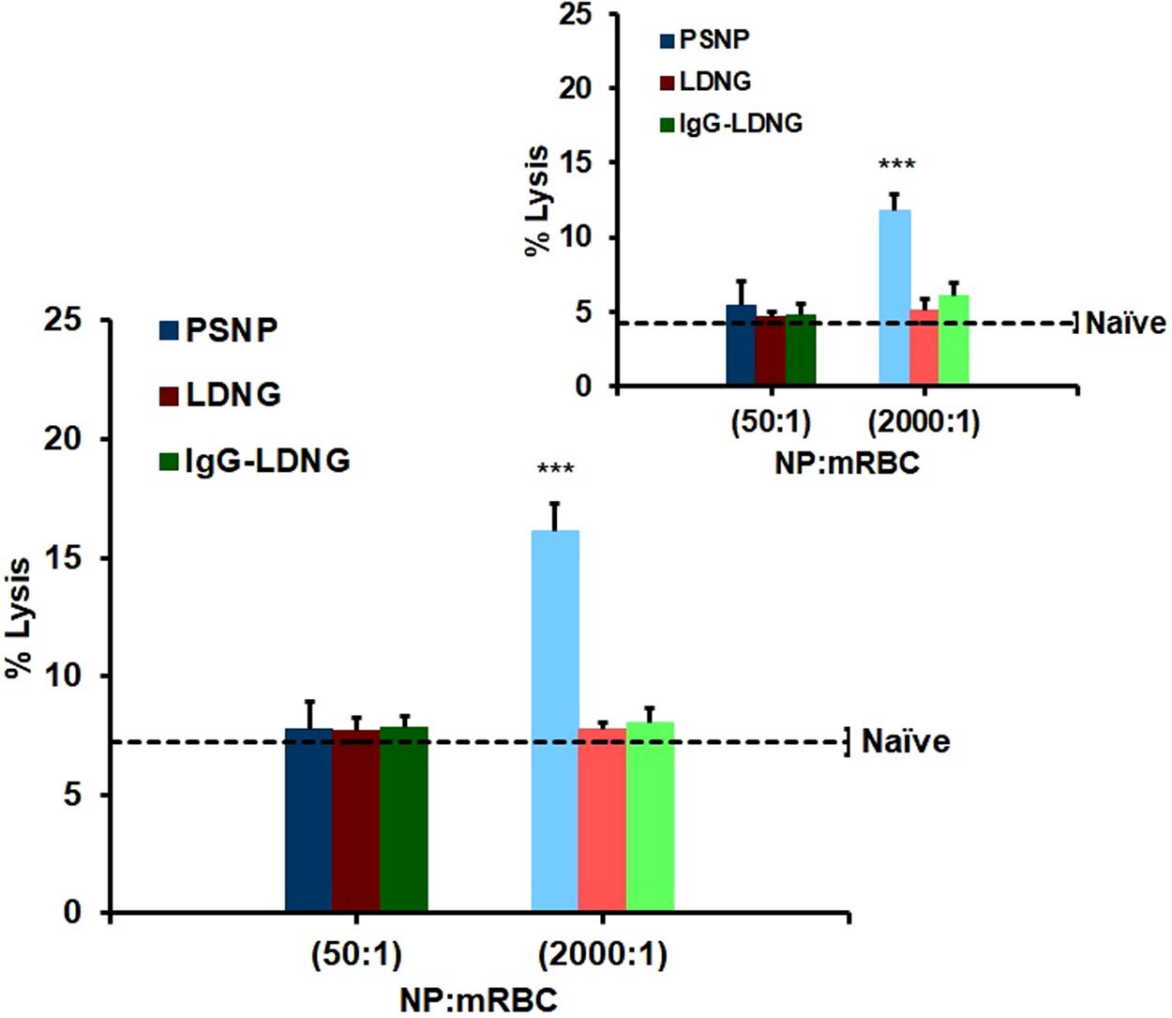
Fragility of murine red blood cells with adsorbed nanoparticles under continuous low mechanical stress: Percent hemolysis for NP:murine RBC ratio of 50:1 or 2000:1 after constant rotation at 37 °C for 8 h. Dotted line represents RBCs without NPs under identical treatment. Inset: Percent hemolysis after 1 h rotation for NP:murine RBCs. Values are means (n = 5) ± SEM (***P < 0.001 vs naïve RBC under low stress).

We examined sensitivity of RBCs suspended in isotonic solution to prolonged moderate mechanical stress caused by rotation at 24 rpm and 37 °C. As shown in Fig. 4, naïve murine RBCs (dashed lines) exhibited low levels of hemolysis at 1 h (4% lysis) and 8 h (7% lysis) rotation. Most likely, this effect reflects deleterious changes in RBCs caused by their isolation, energy starvation, and lack of the stabilizing effects of normal blood plasma. Within the time intervals (up to 8 hours) at which murine RBCs withstood low level shear stresses, PSNP:RBC loading ratios of 200:1 (data not shown) and 50:1 ratios did not sensitize RBCs. However, a PSNP:RBC loading ratio of 2000:1 aggravated hemolysis (12% lysis after 1 h; 17% lysis after 8 h) under low shear stress conditions. Similar exacerbation of lysis was observed for PSNPs of 200 nm and 300 nm diameters (Supplemental Fig. S2). In contrast, LDNGs, both with and without IgG surface coating, did not aggravate mechanical lysis of RBCs, even at the highest tested loading level (2000:1 LDNG:RBC loading ratio). Qualitatively similar results have been obtained with human RBCs, with the exception that the lysis of murine RBC was more extensive than human RBC (Supplemental Fig. S4).

### Sensitivity of NP-carrying RBC to oxidative stress

**Figure 5:**
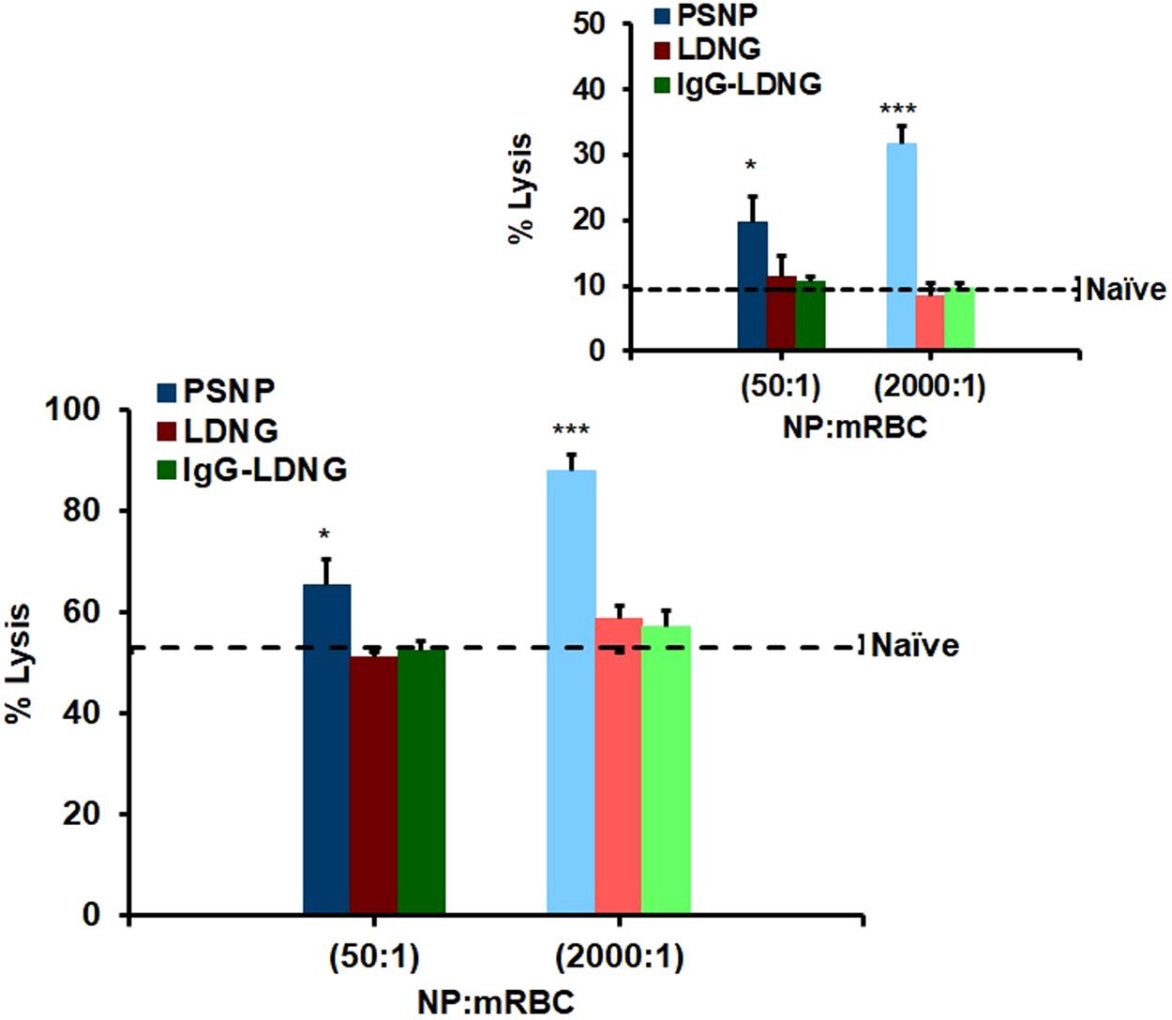
Oxidative fragility of murine red blood cells with adsorbed nanoparticles. Percent hemolysis for NP:murine RBC ratio of 50:1 and 2000:1 after being challenged with H_2_O_2_ under constant rotation for 24 h. Dotted line represents naïve RBC treated with H_2_O_2_. Inset: Percent hemolysis after NP:RBCs challenged 4 h with H_2_O_2_. Values are means (n = 4–5) ± SEM. (*P < 0.05; ***P < 0.001 vs naïve RBC treated with 3 mM H_2_O_2_).

We also assessed the levels of hemolysis induced by H_2_O_2_ under low levels of hydrodynamic stress, following adsorption of NPs. As shown in Fig. 5, 24 h H_2_O_2_ treatment induced 65% and 88% lysis in RBCs loaded with PSNPs at 50:1 and 2000:1, respectively. Under the same conditions, H_2_O_2_ induced 53% lysis in naïve RBCs. Unlike PSNPs, adsorption of LDNGs, both with and without surface conjugation of IgG, onto murine RBCs at LDNG:RBC ratios as high as 2000:1 did not significantly enhance oxidative fragility (58% lysis). We also detected a significant increase in oxidative hemolysis of human RBCs following adsorption of PSNPs (~10-fold over naïve RBCs for 2000:1 PSNPs:RBCs loading). Similar to the murine result, the adsorption of LDNGs at an LDNG:RBC ratio of 2000:1 did not enhance H_2_O_2_-induced hemolysis over results with naïve RBCs (Supplemental Fig. S5).

### Effect of NPs on RBC Exposure of Phosphatidylserine

These data implied that binding of highly-charged PSNP, but not LDNGs, may be detrimental for RBCs at high loading rates. To investigate more specific mechanisms of RBC damage, we determined the effect of NPs on exposure of phosphatidylserine on the surface of the RBCs. Phosphatidylserine mediates the physiological mechanism that marks senescent and damaged RBC for clearance by the RES.

**Figure 6:**
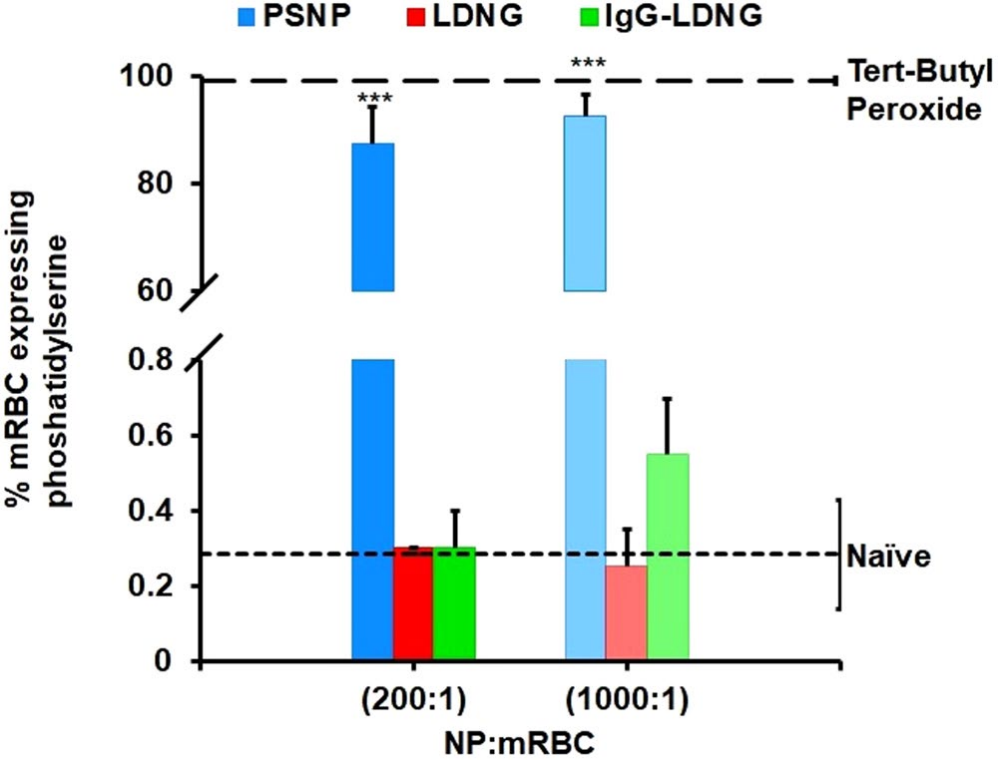
Expression of phosphatidylserine on murine red blood cells. Percentage of phosphatidylserine-exposing murine red blood cells after the adsorption of PSNP, LDNG, or IgG-LDNG at NP:RBC 200:1 or 1000:1 measured by fluorescent annexin V stain with flow cytometry. Tert-Butyl Peroxide was used as a positive control for induction of phosphatidylserine. Values are means (n = 3–4) ± SEM (***P < 0.001 vs naïve RBC).

RBC, PSNP:RBC, LDNG:RBC, and IgG-LDNG:RBC were incubated at room temperature with Annexin V to detect phosphatidylserine. The proportion of phosphatidylserine-exposing murine RBCs dramatically increases after adsorption of PSNP (Fig. 6). At NP:RBC loading ratio of 200:1, PSNP adsorption onto murine RBCs induced an 87% proportion of RBCs expressing phosphatidylserine, compared with 0.1% of naive RBCs and 0.3% of RBCs following LDNG adsorption (0.3%). At NP:RBC ratio of 1000:1, the percentage of murine RBCs expressing phosphatidylserine was 92% for PSNP loading, compared to 0.3% for LDNG loading. Representative flow cytometry scatter plots of murine RBC and NP:RBC exposing phosphatidylserine are shown in Supplemental Fig. S6. Adsorption of PSNP at NP:RBC ratios as low as 200:1 also increased the proportion of human RBCs exposing phosphatidylserine, whereas adsorption of LDNG-based particles at loading ratios as high as 1000:1 did not (Supplemental Fig. S7).

### Effect of bound NPs on RBC deformability

We then used ektacytometry to determine whether or not adsorbed NPs affected RBC deformability. In addition to phosphatidylserine exposure, membrane rigidity is a key determinant of RBC phagocytosis by the RES^67–71^. The ability of RBCs to deform is critical for capillary passage and delivery of oxygen to tissues. Reduced RBC deformability contributes to the pathogenesis of various hematological disorders that impair flow, and augments RBC retention in the spleen^71–78^.

**Figure 7:**
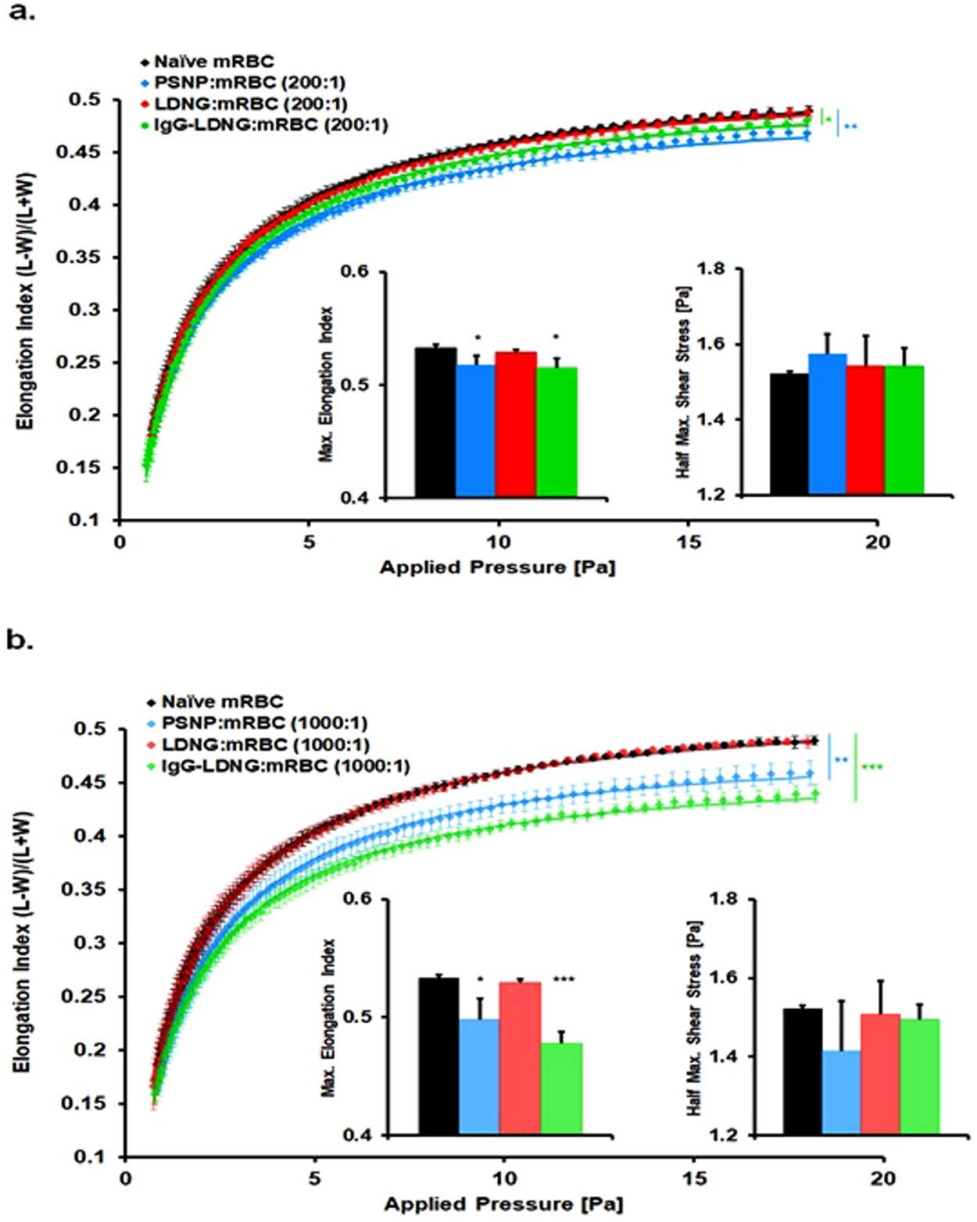
The deformability of murine red blood cells with adsorbed nanoparticles. Elongation index vs shear stress plots of murine red blood cells after adsorption of PSNP, LDNG, and IgG-LDNG at NP:RBC loading ratios of 200:1 (**a**) and 1000:1 (**b**). Values are means (n = 3–5) ± SEM. (*P < 0.05; **P < 0.01; ***P < 0.001 vs naïve RBC).

Our results indicate that the effect of adsorbed PSNP on murine RBCs resulted in a significant dose-dependent decrease in RBC deformability (Fig. 7). The addition of adsorbed PSNP onto RBC at PSNP:RBC ratios as low as 200:1 resulted in a decrease in the EI_max_ value, reflecting the maximum extent to which an RBC can deform, as compared to naïve RBC (0.517 ± 0.008 vs. 0.533 ± 0.003, respectively) (Fig. 7a). PSNP at a high PSNP:RBC ratio of 1000:1 resulted in a further decrease in EI_max_ (0.498 ± 0.017) as compared to naïve RBC counterpart (0.533 ± 0.003). PSNPs of both 200 nm and 300 nm diameter had significant effects on RBC deformability (Supplementary Fig. S2**)**. The adsorption of LDNGs onto murine RBCs at LDNG:RBC loading ratios as high as 1000:1 did not affect the RBC deformability (EI_max_). Surprisingly, the adsorption of IgG-LDNGs resulted in a significant increase in RBC rigidity compared to naïve RBC in a dose-dependent manner; displaying a rightward shift in elongation index curves, resulting in a decrease in EI_max_ values (Fig. 7a and b). In all cases, the SS_1/2_ values, reflecting the rate at which RBCs respond to stress, were not statistically different following NP adsorption for PSNP, LDNG, or IgG-LDNG at any of the investigated NP:RBC ratios.

We also evaluated the deformability of freshly isolated human RBCs from different individuals who have never traveled to malaria-affected areas. Unlike murine RBCs, PSNP adsorption on human RBCs did not affect the RBC deformability (Supplemental Fig. S8**)**. The EI_max_ at NP:RBC loading ratios as high as 1000:1 did not result in a rightward shift in the elongation index curves; Both EI_max_ and SS_1/2_ values remained similar to those for naïve RBC. The adsorption of LDNGs onto human RBCs did not significantly affect deformability up to NP:RBC ratio of 1000:1.

## Discussion and Conclusion

To be clinically useful, nanocarriers/nanoparticles must not induce severe adverse effects on RBCs at loadings necessary for therapeutic applications. This is particularly true for NPs and drug delivery technologies that intentionally interact with the RBC membrane. Previously, we and other labs observed that RBCs may serve as “super-carriers” for NPs, as non-covalent attachment of PSNP to murine RBCs markedly alters the biodistribution of the NP in a manner advantageous to treatment of many diseases. In these seminal studies, PSNPs were used as a representative NP to evaluate RBCs as cellular carriers for NP delivery. However, in our subsequent studies, it has been shown that bare PSNPs induce osmotic, mechanical, and oxidative hemolysis as well as RBC agglutination at PSNP:mRBC loading ratios 50:1 or greater^48^. While this as such is not a concern since PSNPs are simply a model particle and not a therapeutic vehicle, this observation brings up the question about engineering of particle properties to mitigate RBC toxicity. Agglutination is possibly due to the highly charged surface of PSNP as compare to LDNG; the more negatively charged PSNP surface may bridge RBCs in in suspension via electrostatic interactions.

In our current work, we show that the adsorption of PSNP in high loadings has additional detrimental effects on RBCs including 1) promoting the exposure of phosphatidylserine (a marker for cell senescence) on the surface of RBCs and 2) increasing the rigidity of the RBC. PSNPs of both 200 nm and 300 nm diameter induced RBC agglutination at loading concentrations over PSNP:RBC of 50:1 and enhanced RBC sensitivity to osmotic and mechanical stress. We also show that some of the detrimental effects caused by bare PSNP can be circumvented by conjugating antibodies to the surface of the bare PSNP. This indicates that the observed results are due to the physicochemical properties of polystyrene; a terminal layer of IgG will change the surface charge and elasticity of the PSNP which. For example, the surface charge of IgG-PSNP is closer to neutral compared to PSNP, suggesting minimizing surface charge might prevent RBC agglutination and alter the interactions of PSNPs with the RBC membrane.

Since it is widely accepted that material properties of NPs influence their interactions with cells (e.g. attachment, uptake, toxicity, and compatibility), studies focused on elucidating the effect of material properties on carrier RBCs will direct future translational efforts^64^. In the present study, we have taken these first steps towards translational efforts by utilizing biodegradable LDNGs as opposed to non-biodegradable PSNPs. LDNG was chosen as a second nanoparticle in this study because its properties contrast those of PSNPs in a number of critical ways; LDNGs are less rigid compared to PSNPs, the components of LDNGs are biodegradable and have demonstrated biocompatibility in previous work^66^, and the surface chemistry of LDNGs is characterized by strong hydrophilicity and minimal surface charge. Furthermore, there have been studies that have shown LDNGs, loaded with dexamethasone, can be useful for drug delivery^66^.

We have demonstrated that LDNGs do not induce NP-mediated damage (increased membrane stiffness, increased agglutination, etc) to RBCs at loadings at which PSNPs induce adverse effects. Importantly, this was observed for LDNGs even at NP:RBC loading ratios up to 2000:1. Key physicochemical differences between LDNGs and PSNPs are likely responsible for the observed differences in their RBC sensitization and compatibility; LDNGs with antibody conjugated to their surface, which still have a zeta potential close to neutral, also didn’t induce toxic effects on RBCs, further supporting the hypothesis that near neutral surface charge correlates with NP-RBC compatibility. Even though the mechanism of LDNG-RBC compatibility has not been fully elucidated, we believe that the composition and surface chemistry of NPs are major factors in their biocompatibility with RBCs.

For example, PSNPs are rigid and hydrophobic, non-responsive to aqueous environments, and have highly charged surfaces; PSNPs may alter the organization of RBC membrane phospholipids, leading to phosphatidylserine exposure, resulting in loss of integrity, and possibly causing pore formation in the RBC membrane leading to lysis. However, the conjugation of antibody to the surface of the rigid PSNP might prevent the rigid material from directly interacting with the RBC membrane. On the other hand, LDNGs are soft, swellable, hydrophilic networks and wouldn’t cause these detrimental effects; The deformable surface of the LDNGs would facilitate flexible contact points between the RBC and LDNG surfaces^29^ and the near-neutral surface charge of LDNGs is less likely to initiate electrostatic interactions that can lead to disruption of RBC membranes and/or surface proteins. Furthermore, hydrophilicity of the surface and internal hydrogel network of LDNGs allows the LDNGs to take on key properties of the fluid osmotic environment in which LDNG-RBC attachment, circulation, and delivery occur.

Although few, if any, harmful effects of LDNG attachment to RBCs were observed *in vitro*, further studies are necessary to justify the use of LDNGs in RBC drug delivery. In previous studies, LDNGs have been successful in delivering drugs, and RBCs have been used as carriers for NP delivery. However, additional toxicological studies should be performed to confirm biocompatibility of LDNGs with other cell types^66^. Future research utilizing carrier RBCs to deliver LDNGs will be focused on elucidating toxicological effects of the adsorbed LDNGs *in vivo*. In conclusion, we have extended our understanding of NP-RBC delivery systems by highlighting how the material properties of NPs can be used to control interactions with the carrier RBC through limiting negative effects to the carrier cell. Although NPs were adsorbed onto isolated RBCs, and the NPs might interact with various components in the blood when delivered *in vivo*, these results illustrate the potential utility of LDNGs in treating diseases by delivery of a greater drug payload when adsorbed onto RBCs.

## Materials and Methods

### Ethics Statement

All animal studies were carried out in strict accordance with Guide for the Care and Use of Laboratory Animals as adopted by the National Institutes of Health, and approved by the University of Pennsylvania Institutional Animal Care and Use Committee (IACUC). All experiments were performed in accordance with relevant guidelines and regulations. Mice were anesthetized with ketamine/xylazine. All animals were euthanized by cervical dislocation and death was confirmed by cessation of heartbeat.

All studies involving human subjects were approved by the University of Pennsylvania Institutional Review Board. Written informed consent from donors was obtained for the use of blood samples in this study. Blood samples were destroyed after the study. Names and any personal information about individual participants were not taken.

### Blood Collection

CJ7BL/6 J male mice were purchased from the Jackson Laboratory (Bar Harbor, ME). All mice were housed in a temperature- and humidity-controlled environment (18–23 °C with 40–60% humidity under a 12-hour light-dark cycle) with ad libitum access to food (Labdiet 5010 autoclavable rodent diet, Brentwood, MO) and water.

Blood from CJ7BL/6 J mice was harvested at the University of Pennsylvania. Whole blood was collected in EDTA to prevent coagulation. Blood donation of human voluntary donors also occurred at the University of Pennsylvania. A volume of 4 mL of whole blood was collected in a vial containing ~3.2% Na citrate (BD Vacutainer). Blood was centrifuged at 1000 g for 10 min at 4 °C to remove plasma and buffy coat and isolate erythrocytes.

Isolated erythrocytes (RBCs) were washed by adding ice cold 1× Dulbecco’s-Phosphate-Buffered-Saline (DPBS), pH 7.4, up to 12 mL total volume and pipetting gently up and down to mix buffer with RBC extensively. The RBC suspension was centrifuged again (500 g, 15 min, 4 °C) and the supernatant was discarded. This wash step was repeated a total of 3 times.

### Synthesis of Lysozyme Dextran Nanogels

LDNGs were synthesized via a two-step heating process^65^. Briefly, dextran and lysozyme were dissolved in a 1:1 mol:mol ratio in water. The pH of the mixture was adjusted to ~7.1 and the mixture was lyophilized. The resulting powder was allowed to react at 60 °C at ~80% relative humidity for 24 h over saturated KBr solution. The reacted lysozyme and dextran was dissolved in water at 5 mg/mL and the solution pH was adjusted to 10.7, prior to reaction for 30 min at 80 °C. The resulting LDNGs were stored at 4 °C.

### Conjugation of IgG onto Nanoparticles

EDC carbodiimide crosslinker chemistry was used to conjugate rat IgG onto polystyrene beads Briefly, PSNP solutions were buffer exchanged with 50 mM MES pH 5.2. Sulfo-NHS was added to a concentration of 0.275 mg/mL and PSNP-Sulfo-NHS solutions were incubated for 3 min. EDAC was then added to a final concentration of 0.1 mg/mL and the solution was incubated for 15 min. IgG was added at 200 IgG per bead and the solution was incubated for 4 h on a shaker at low speed. Thereafter, IgG-PS beads were spun down at 12,000 g and resuspended in PBS. IgG-PS were then sonicated at 30% amplitude 3 times.

The attachment of IgG to LDNGs was performed as previously reported^66^. Briefly, unmodified LDNGs were oxidized by incubation with sodium periodate (2.5 mM) at room temperature for 3 days in the dark. IgG was then added to the LDNGs in DI water and the IgG-LDNG mixture was incubated at 4 °C overnight. Following the conjugation of IgG via amine-aldehyde bond, IgG-LDNGs were centrifuged at 16,000 g for 15 min and supernatant containing unbound IgG was removed. IgG-LDNG pellet was then re-suspended into PBS.

### Nanoparticle Radiolabeling

Both PS beads and LDNGs were radiolabeled with ^125^I-IgG with the methods used above. Unmodified LDNGs and IgG itself were directly radiolabeled with ^125^I using Pierce iodination beads. Unmodified PSNP were radiolabeled with ^3^H conjugated oleic acid as previous described^29^.

### Adsorption of Nanoparticles to RBCs

Briefly, murine or human RBCs were incubated with either unmodified or IgG-coated nanoparticles (NPs) (200 nm and 300 nm carboxylated polystyrene beads (PSNP) or 268 nm lysozyme-dextran nanogels (LDNG)) at NP:RBC ratios of 50:1, 200:1, 1000:1, or 2000:1 for 1 h under constant rotation at 4 °C. NP:RBC solutions were washed with PBS three times at 100 g for 8 min to remove non-adsorbed NPs prior to experiments.

For direct comparison with other particles, such as IgG-PSNP at a NP:RBC ratio of 200:1, we used linear regression to estimate the value of the adsorption efficiency for PSNP:RBC at that ratio. The linear regression model used 4 data points over a range of NP:RBC ratios of 50:1 to 2000:1, yielding an r-squared value of 0.99 and an estimated adsorption efficiency of 24% for a PSNP:RBC ratio of 200:1.

The number of RBCs in 10% hematocrit solutions was determined based on theoretical RBC volume. The number concentrations of PSNPs in stock solutions were provided by the manufacturer. LDNG concentrations in stock solutions were set by using standardized mass concentrations of lysozyme and dextran during LDNG synthesis and drawing LDNGs used in the RBC studies from the original LDNG stocks as synthesized. After radiolabeling the NPs, the initial volume of NPs (either PSNPs or LDNGs) added to RBCs was chosen according to the desired NP:RBC loading ratio. The final quantity of NPs loaded on RBCs (and efficiency of NP loading) was determined by tracing radioactivity on pelleted RBCs and comparing to initially introduced levels of NP-associated radioactivity.

### RBC Osmotic Fragility

Osmotic fragility assay on freshly-obtained RBC and NP:RBC was performed as previously reported^48^. Briefly, washed RBC and NP:RBC suspensions were placed in salt concentrations ranging from 0 mM to 150 mM at 37 °C. NP:RBC or RBC suspensions, at a final concentration of 1% hematocrit, were then immediately centrifuged at 13,400 g for 4 min and the absorbance of the supernatant was recorded at 540 nm by SpectraMax M2 plate reader (Molecular Devices) as an indicator of hemoglobin release due to RBC lysis. Hemoglobin release following RBC submersion in water was taken as 100% RBC lysis.

### RBC Fragility under low shear stress

Low shear stress fragility assay was performed on freshly-obtained washed RBCs. All RBC samples were subjected simultaneously to the same stress. RBC and NP:RBC suspensions of 1.0% hematocrit in DPBS were rotated (24 rpm) for different time periods at 37 °C. RBC and NP:RBC suspensions were centrifuged at 13,400 g for 4 min, supernatants were obtained, and hemoglobin in the supernatant was immediately measured by absorbance at 540 nm with a SpectraMax M2 plate reader (Molecular Devices). Hemoglobin release following RBC submersion in water was taken as 100% RBC lysis.

### RBC Oxidative Fragility under low shear stress

Freshly-obtained RBCs were subjected to hydrogen peroxide-induced lysis. Identical oxidative stress was simultaneously applied to all erythrocyte samples. 1.0% hematocrit suspensions of RBCs and NP:RBCs, with 3 mM H_2_O_2_ in DPBS, were rotated (24 rpm) for different time periods at 37 °C. Control samples were not subjected to H_2_O_2_ treatments. The hemoglobin released from the RBCs during rotation was measured, as was the free hemoglobin in the control samples, by 540 nm absorbance with a SpectraMax M2 plate reader (Molecular Devices). Hemoglobin release following RBC submersion in water was taken as 100% RBC lysis.

### RBC Phosphatidylserine

Phosphatidylserine exposure on RBCs was measured according to previously reported flow cytometry procedure based on the binding of Annexin V-Alexa Fluor^TM^ 488 (Invitrogen) to phosphatidylserine. Briefly, RBC and NP:RBC suspensions at 0.01% hematoocrit were incubated at room temperature with fluorescent annexin V in buffer containing 2 mM CaCl_2_ for 15 min. After incubation, an aliquot was aspirated directly into the Accuri Flow Cytometer C6 (BD) for analysis. RBC populations were defined by forward and side scatter parameters. Results were expressed as percentages of phosphatidylserine-positive RBCs. Each experiment was repeated at least three times, and statistical analysis was performed.

### RBC Deformability

RBC and NP:RBC deformability was measured by ektacytometry (Rheo Meditech, South Korea) operating at room temperature. Briefly, 25 µL of 10% hematocrit suspension of RBCs or RBC:NP was mixed with 675 µL of 5.5% (w/v) 360 kDa polyvinylpyrrolidone (Sigma Aldrich). RBC suspensions were placed into flow channels and subjected to shear stress varying from 0 Pa to 18 Pa. Ellipsoidal diffraction patterns generated by RBC suspensions under stress were recorded and relationship between applied stress and elongation of the diffraction index was analyzed. Modeled maximum elongation index (EI_max_) and half maximum shear stress (SS_1/2_) were determined. Each experiment was repeated at least three times.

### Statistical Analysis

Results are expressed as mean ± SEM unless otherwise noted. Significant difference between means were determined by two tailed t-test. P < 0.05 was considered statistically significant.

### Data availability

All data generated or analyzed during this study are included in this published article (and its Supplementary Information files).

## Electronic supplementary material

Supplementary Information
