# Supplementary material for: Nanoparticle Properties Modulate Their Attachment and Effect on Carrier Red Blood Cells
Source: Sci Rep. 2018 Jan 25;8:1615. doi: 10.1038/s41598-018-19897-8 (PMC5785499; doi:10.1038/s41598-018-19897-8)
Supplement: Supplementary file 1 — Supplementary Information [file 41598_2018_19897_MOESM1_ESM.doc]

**Nanoparticle Properties Modulate Their Attachment and Effect on Carrier Red Blood Cells**

**Daniel C. Pan, Jacob W. Myerson, Jacob S. Brenner, Priyal N. Patel, Aaron C. Anselmo, Samir Mitragotri, and Vladimir Muzykantov**

**
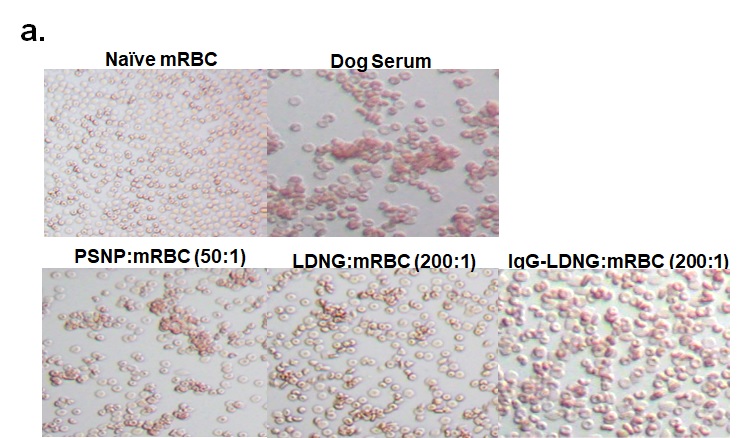
**

**
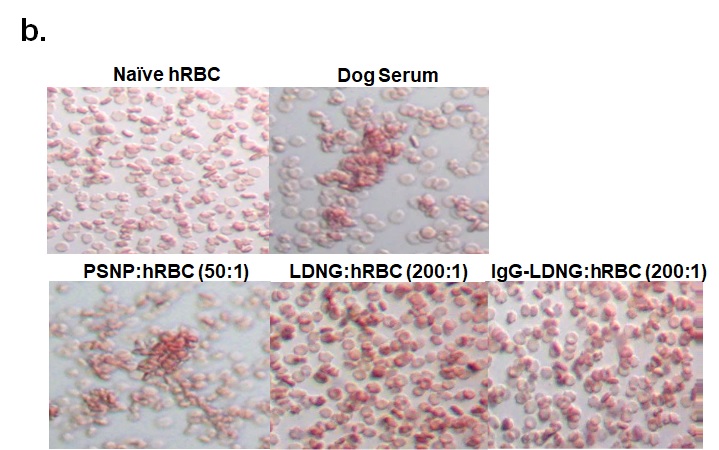
**

**Supplemental Figure 1: Red blood cells agglutination with nanoparticles adsorbed onto their surface.** Agglutination was visualized using a light microscope at 250x magnification for murine (a) and human (b) red blood cells. Dog serum was used as a positive control.


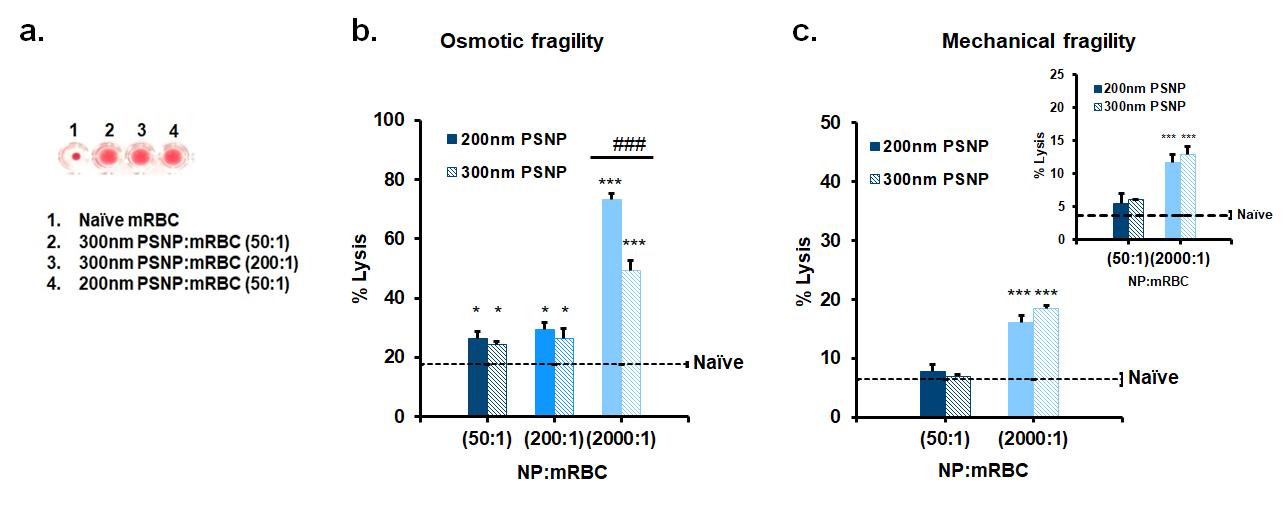


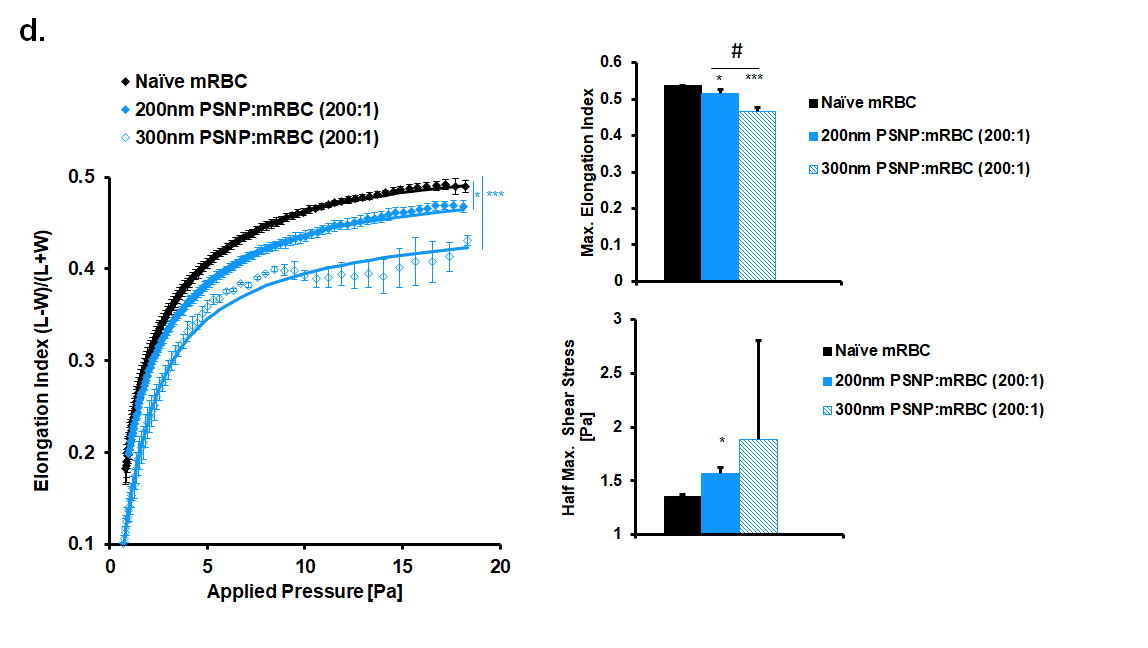


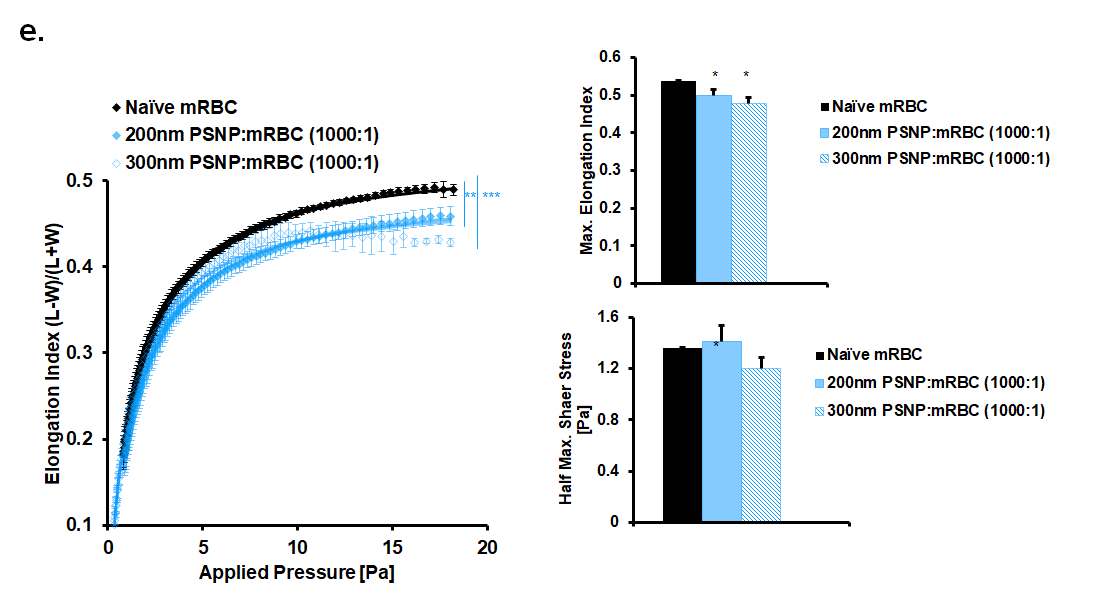


**Supplemental Figure 2: Biocompatibility of murine red blood cells with different sized PSNP adsorbed onto their surface.** Agglutination was visualized using U-shaped bottom plate (a), percent hemolysis of different RBC:NP/RBC ratios after immediate exposure at 73mM NaCl (b), percent hemolysis of RBC:NP/RBC ratio of 50:1 and 2000:1 after constant rotation at 37C for 8h. Inset: percent hemolysis after 1h RBC:NP/RBC ratio (c). Elongation index vs shear stress plots of RBC:NP/RBC loading ratios of 200:1 (d) and 1000:1 (e). Dotted lines represent naïve RBC under osmotic or low stress conditions. Values are means (3-6) ± SEM. (*, P<0.05; ***, P<0.001 vs naïve RBC; #, P<0.05; ###, P<0.001 vs 200nm PSNP).

**
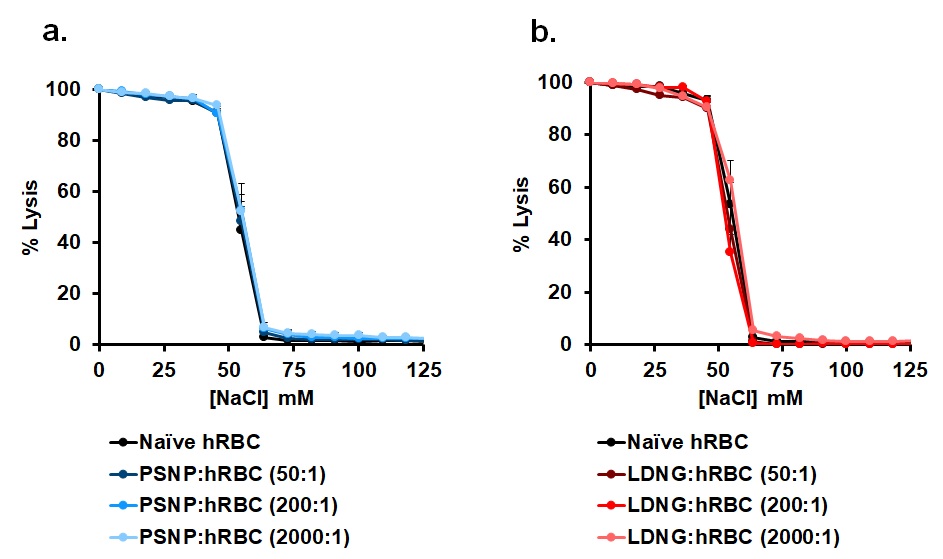
**

**
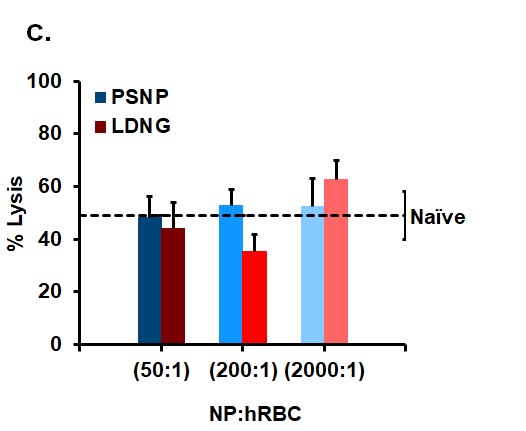
**

**Supplemental Figure 3: Osmotic fragility of human red blood cells with adsorbed nanoparticles.** Representative osmotic fragility curves for human RBC:NP/RBC at a NP:RBC of 50:1, 200:1, and 2000:1 after immediate exposure to different [NaCl] after adsorption of PSNP (a) and LDNGs (b). Percent hemolysis of different RBC:NP/RBC ratios at 55 mM NaCl (c). Values are means (n=4-6) ± SEM.

**
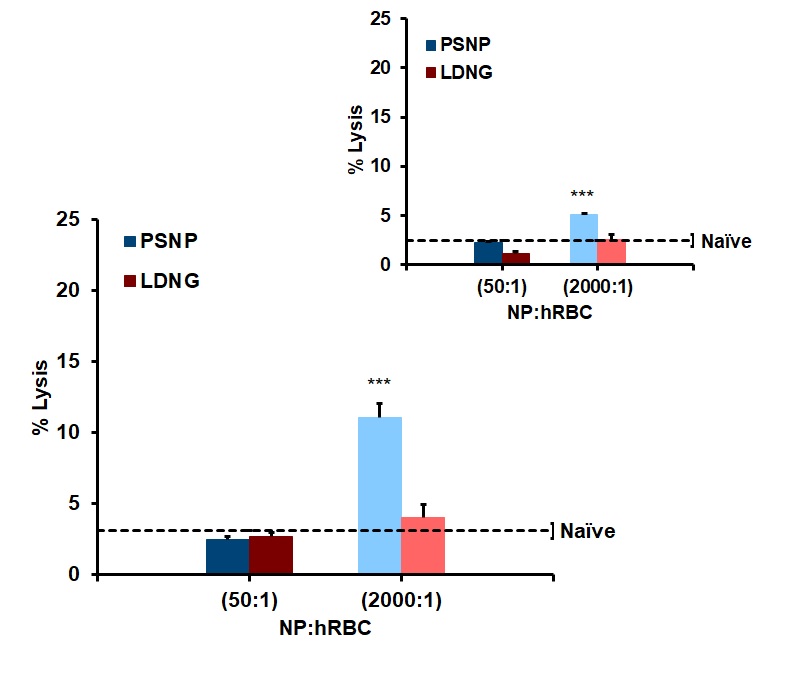
**

**Supplemental Figure 4: Fragility of human red blood cells with adsorbed nanoparticles under continuous low stress:** Percent hemolysis of human RBC:NP/RBC ratio of 50:1 and 2000:1 after constant rotation at 37ºC for 8 h. Dotted line represents naive RBC under continuous low stress. Inset: Percent hemolysis after 1h for human RBC:NP/RBC. Values are means (n=5) ± SEM (***, P<0.001 vs naïve RBC under low stress).


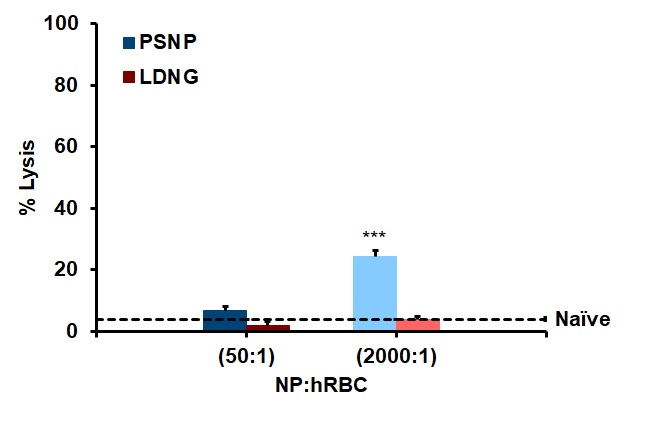


**Supplemental Figure 5: Oxidative fragility of human red blood cells with adsorbed nanoparticles.** Percent hemolysis of human RBC:NP/RBC ratio of 50:1 and 2000:1 after being challenged with H_2_O_2_ under constant rotation for 24 h. Dotted line represents naïve RBC treated with H_2_O_2_. Values are means (n=4) ± SEM. (***, P<0.001 vs naïve RBC treated with 3 mM H_2_O_2_).


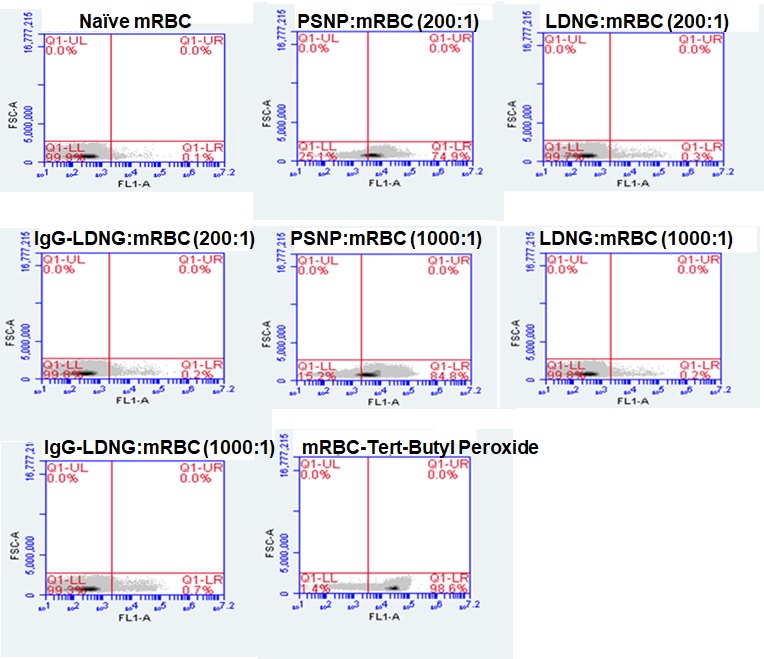


**Supplemental Figure 6: Percentage of murine red blood cells expressing phosphatidylserine.** Representative density plots murine red blood cells expressing phosphatidylserine after the adsorption of PSNP, LDNG, and IgG-LDNG at a NP:RBC (200:1) and (1000:1) measured by fluorescent annexin V on flow cytometry. Quadrants indicate cut offs for determining phosphatidylserine exposing red blood cells. Tert-Butyl Peroxide was used as a positive control. Values are means (n=3-4).

**
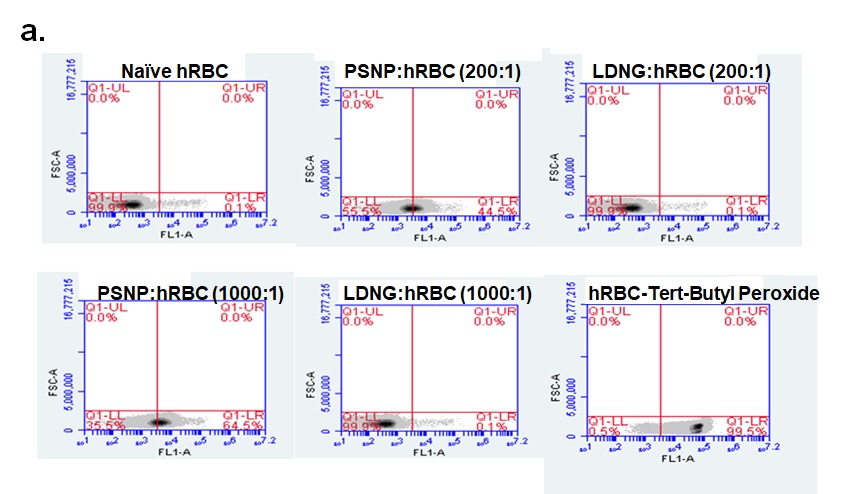
**

**
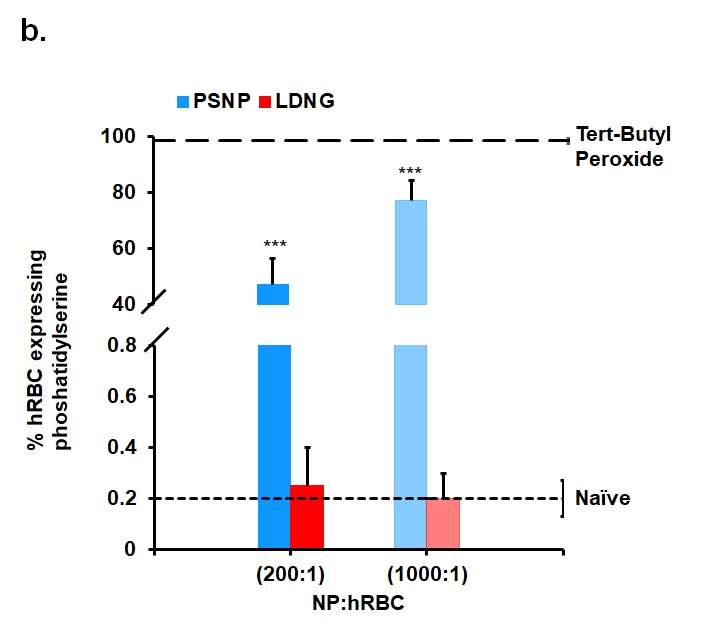
**

**Supplemental Figure 7: Expression of phosphatidylserine on human red blood cells.** Representative density plots (a) and graph (b) of phosphatidylserine exposing human red blood cells after the adsorption of PSNP and LDNG at a NP:RBC (200:1) and (1000:1) measured by fluorescent annexin V on flow cytometry. Quadrants indicate cut offs for determining phosphatidylserine exposing red blood cells. Tert-Butyl Peroxide was used as a positive control. Values are means (n=3) ± SEM (***, P<0.001 vs naïve RBC).


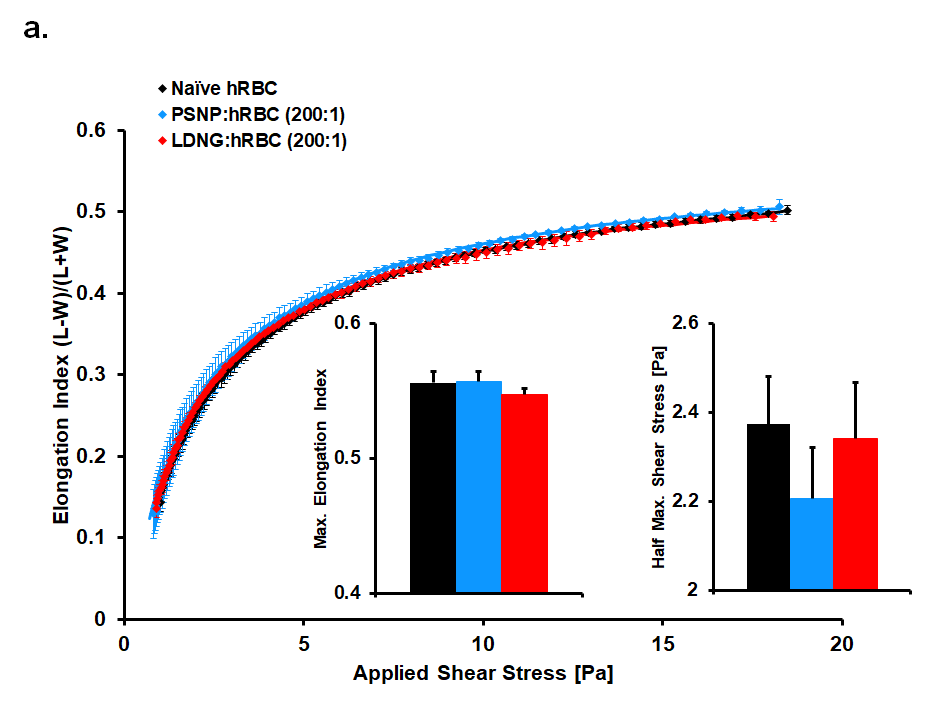


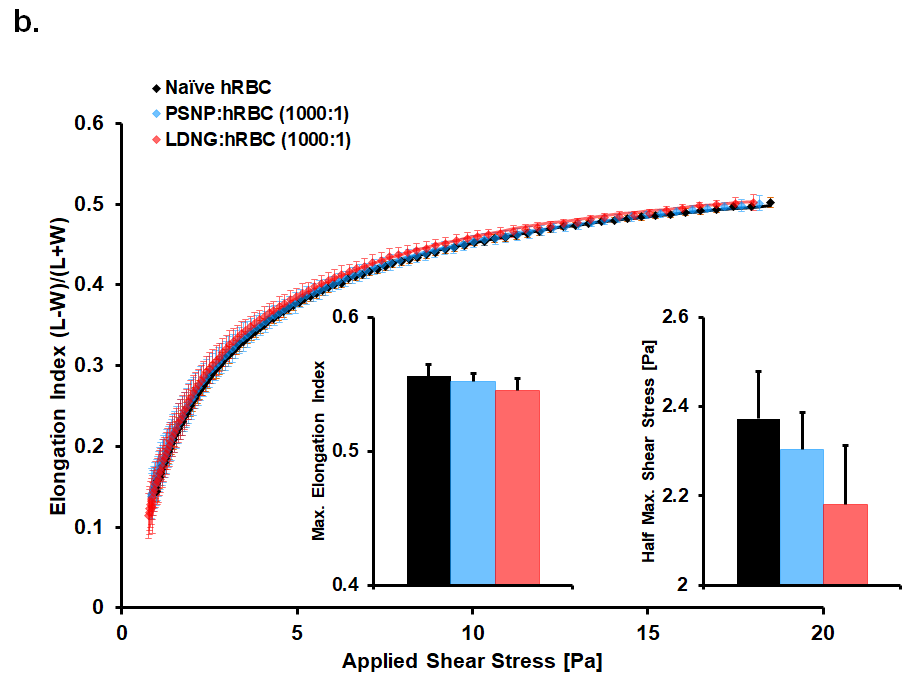


**Supplemental Figure 8:** The deformability of human red blood cells with adsorbed nanoparticles. Elongation index vs shear stress plots of human red blood cells after the adsorption of PSNP and LDNG at RBC:NP/RBC of 200:1 (a) and 1000:1 (b). Values are means (n=4-5) ± SEM.

**Supplemental Table 1:** Size and charge of various sized PSNP.

|  | 200nm PSNP | 300nm PSNP |
| --- | --- | --- |
| Size (nm) | 171.1 ± 3.0 | 319.0 ± 1.6 |
| PDI | 0.023 ± 0.01 | 0.069 ± 0.02 |
| Zeta Potential (mV) | -33.27 ± 1.05 | -54.83 ± 0.23 |
